# The influence of crossbite in early development of mandibular bone asymmetries in paediatric patients

**DOI:** 10.4317/jced.54110

**Published:** 2017-09-01

**Authors:** Montserrat Diéguez-Pérez, M. Joaquín de Nova-García, Mª Rosa Mourelle-Martínez, Cristina González-Aranda

**Affiliations:** 1 Departamento de Odontopediatría, Profilaxis y Ortodoncia. Facultad de Odontología Universidad Complutense de Madrid, Plaza Ramón y Cajal, 3 - Ciudad Universitaria, 28040 Madrid

## Abstract

**Background:**

All authors agree that posterior crossbite is a malocclusion that affects mandibular growth and may lead to skeletal asymmetry but there are few data on which age these modifications are easily quantifiable.

**Material and Methods:**

For this study, the researchers used x-ray records of 217 children between 6 and 9 years of age, in the mixed dentition stage and with unilateral posterior crossbite. All the horizontal variables were traced and evaluated by the principal researcher, using the tpsDig version 2 computer program. Subsequently, a descriptive and statistical analysis was carried out, using the SPSS 17.0 for Windows program.

**Results and Discussion:**

After analysing the vertical mandibular traces on the x-rays, the researchers found, in all cases, quantifiable differences between the crossbite side and the non-crossbite side. The differences between horizontal variables were statistically significant (*p*<0.005) for the entire sample (H3-H4), in the group of boys (H3-H4) and in the 7-year old age group (H1-H2 and H3-H4). Differences were observed in the size of the horizontal measures between the crossbite side and the non-crossbite side. Some of these differences were significant as a function of the sex and age of the study sample.

** Key words:**Crossbite, Mandibular asymmetry, Panoramic.

## Introduction

Patients with unilateral crossbite present occlusal, postural and functional alterations, which can lead to a reduction in bite force, asymmetrical muscular activity, joint problems and changes, not only in the position of the mandible, but also in its movements. If this pathology persists throughout the patient´s growth, it facilitates the development of skeletal asymmetry (1).

There are different opinions about the manner in which this malocclusion affects mandibular growth and the development of skeletal asymmetry. Some researchers have observed that a unilateral posterior crossbite at an early age leads to bone development of an asymmetrical mandibular ramus with larger vertical dimensions. They have frequently observed, on the crossbite side, the presence of a condyle in a more posterior position (2-5).

Different authors have tried to evaluate and determine the precision of images and measurements of the mandible using orthopantomography (6-10). They have also studied the possibility of using these images as an aid in diagnosing pathologies such as hyperplasia of the coronoid process and condylo-mandilbular asymmetry, among others (10-16).

Orthopantomography has also been used to determine the patient’s skeletal pattern and mandibular foramen (17,18). Currently, there is no consensus among authors regarding the precision of measurements using digital orthopantomography (6,18).

It is therefore not known at what age skeletal asymmetry provokes quantifiable bone changes in paediatric patients. We analysed panoramic x-rays of patients at early ages, in order to evaluate bone development, keeping in mind the crossbite side.

To evaluate possible alterations in bone development that may be caused by malocclusion, by studying panoramic x-rays of a sample of children with unilateral crossbite. At the same time, to verify through these routine examinations at an early age whether any bone changes occurring are quantifiable, whether they increase with the age of the patient, and whether they are observed equally in both sexes.

## Material and Methods

The study sample consisted of 645 patients attending a Diagnostic Radiology Centre over the last five years. All the children were recruited randomly. Afterwards, photographic and radiographic (orthopantomographic) records were taken. General medical data were recorded. The parents or guardians of all the children signed an informed consent form authorising the use of the records for research purposes, in compliance with the Data Protection Law.

Subsequently, the following criteria were applied for inclusion:

• Healthy patients, in first-phase mixed dentition, with a unilateral posterior crossbite.

• No history of having received corrective treatment for malocclusion.

• Photographic records that allow for precise diagnosis of the malocclusion under study.

• Panoramic x-rays of sufficient quality to be evaluated.

And for exclusion:

• Patients with any orofacial pathology, dysmorphology, or syndrome that might cause alterations in normal development and/or growth.

• The presence of dental/periodontal alterations that might affect or interfere with the diagnosis of crossbite.

• Patients who use orthodontic appliances.

After applying these criteria, the selected sample consisted of 217 children between 6 and 9 years of age. The median age of the sample was 7.5 years.

The photographic diagnosis protocol included a series of digital photographs taken by the examiner himself, meeting the same technical requirements. The photographic series included extraoral projections while at rest (front, profile and three-quarters), and intraoral (front, right and left sides and submentonian) in occlusion and at maximum opening (occlusal, maxillary and mandibular). The photographs were evaluated by the principal researcher using a computer (with a 30-inch screen), for a maximum of 20 patients per session, and the image was magnified when required. The following variables were taken into account: the number of teeth involved, the deviation from the median line, and the type of anterior and/or posterior crossbite.

Likewise, all the x-rays were taken using the same technical specifications, and the verbal instructions were the same for all the patients. The x-rays were again examined by the principal researcher. In case of duplication, the best-quality photograph was selected. All the photographs were analysed using a computer and a 30-inch monitor, as well as the software programme tpsDig version 2. A maximum of 20 x-rays were examined in each session. Initially, the digital image from the x-ray was captured and a zoom was used to enlarge or reduce it by 10% each time, facilitating anatomical recognition of structures of interest. The anatomical points for the study were then located. Finally, the traces and estimates of horizontal measurements were made, at the level of the mandibular body:

Variable H1: Distance between the most protruding and highest point of the right condyle and the perpendicular V0.

Variable H2: Distance between the highest, most protruding point of the left condyle and the perpendicular V0.

Variable H3: Distance between the highest, most protruding point of the right coronoid apophysis and the perpendicular VO.

Variable H4: Distance between the highest, most protruding point of the left coronoid apophysis and the perpendicular VO.

Variable H5: Distance between the bisector of the right mandibular angle and the perpendicular VO.

Variable H6: Distance between the bisector of the left mandibular angle and the perpendicular VO.

The measurements obtained were subjected to statistical analysis using the SPSS 17.0 for Windows program. A linear model was employed to determine the inter-subject factors. A Student’s T-Test was applied to each of the measurements individually, comparing at all times the side that had the crossbite with the non-crossbite side. The median, standard deviation and N value for each of the measurements in the total sample were found. In each of the results, a check was made to see whether there were significant differences at 95% (*p*< 0.05). Post hoc tests were also performed to determine the differences between the medians for each age range and for each of the measurements. Following the last measurement, after 20 days had passed, the principal researcher randomly selected 20% of the total measurements, to take them again. A paired T test was applied to detect systemic error.

## Results

Upon studying the length of the horizontal variables in the entire sample with right unilateral posterior crossbite (RUPC), greater length was found in variables H2, H4 and H6, corresponding to the left side, compared to variables H1, H3 and V5 on the right side. All the horizontal estimations were always greater on the side without malocclusion. These results showed that only the differences between the horizontal variables H3-H4 were statistically significant.

Analysis of the lengths of the horizontal variables in the sample of boys with RUPC reflected greater length in the H2, H4 and H6 variables corresponding to the left side, compared to the H1, H3 and V5 variables on the right side. Once again, as in the total sample, there was only significance in the difference between the left and right horizontal variables H3-H4.

Analysis of the lengths of the horizontal variables in the sample of girls with RUPC showed greater length in the H1 and H5 variables corresponding to the right side, compared to the H2 and H6 variables on the left side. In this study group, the length of the H3 variable on the right side was less than the H4 variable on the left side, just as in the total sample, and in the boys. On this occasion, no statistically significant differences were observed in any of the variables (H1-H2, H3-H4 and H5-H6).

Analysis of the lengths in the age 6 sample showed greater length in the H2, H4 and H6 variables on the side without malocclusion, compared to the H1, H3 and H5 variables on the side with malocclusion. No statistically significant differences were found in any of the differences between variables (H1-H2, H3-H4 and H5-H6). Analysis of these same lengths in the age 7 sample also showed greater length in the H2, H4 and H6 variables on the side without malocclusion, compared to the H1, H3 and H5 variables on the side with malocclusion. In this age range, statistically significant differences were observed between the H1-H2 and H3-H4 study variables. Analysis of horizontal lengths in the age 8 sample showed greater length in the H2 and H4 variables on the side without malocclusion; however, greater length was observed in the H5 variable on the side with malocclusion. None of the differences between variables were significant in this group. Finally, analysis of the length of the study variables in the age 9 sample found greater lengths for the H1 and H5 variables on the side with malocclusion. No statistically significant differences were found in any of the differences between variables (H1-H2, H3-H4 and H5-H6) ( Table 1, Table 2).

**Table 1:**
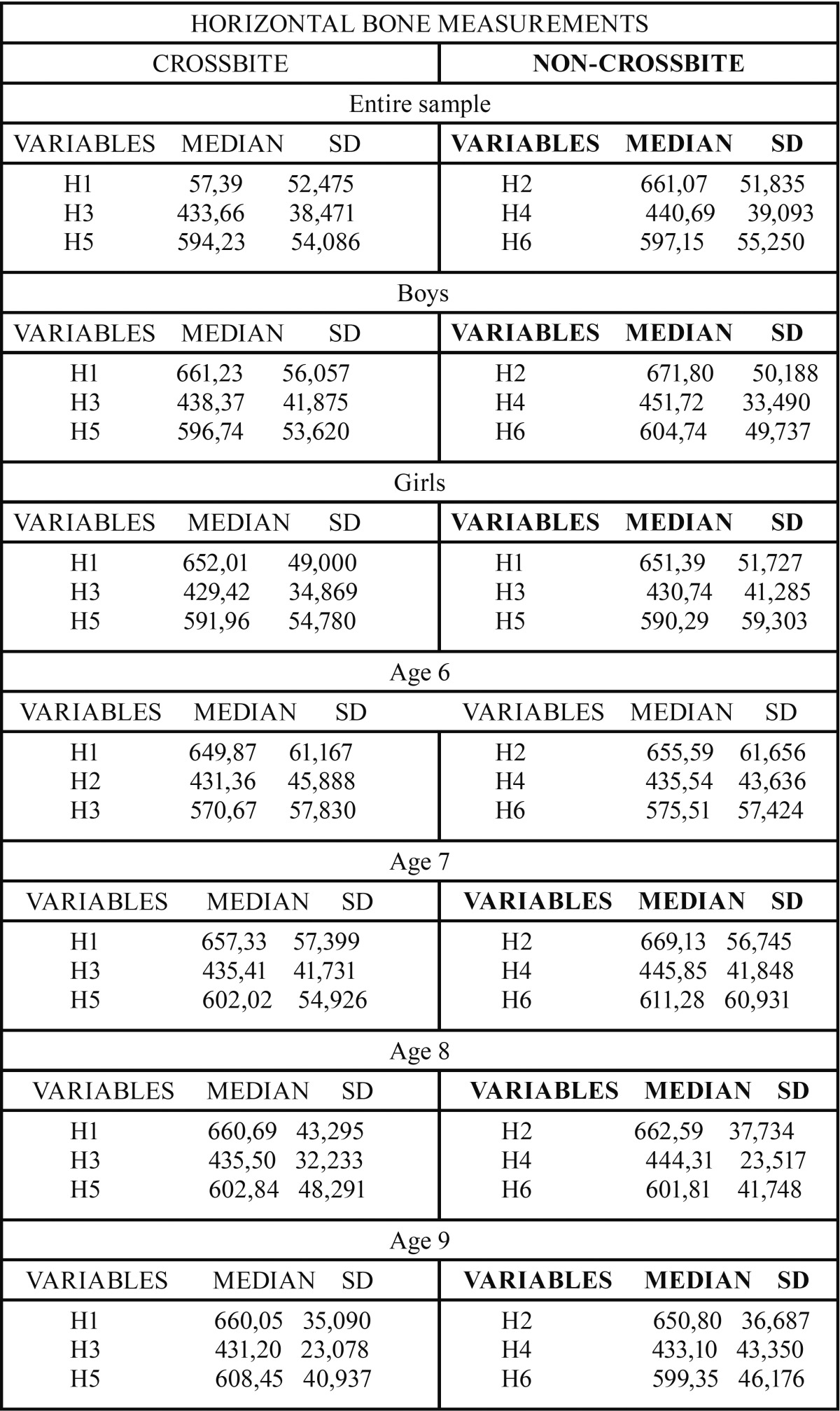
Horizontal bone measurements for the entire sample, by sex and age, in paediatric patients with Unilateral Posterior Crossbite (UPC). Median; S.D: Standard Deviation.

**Table 2:**
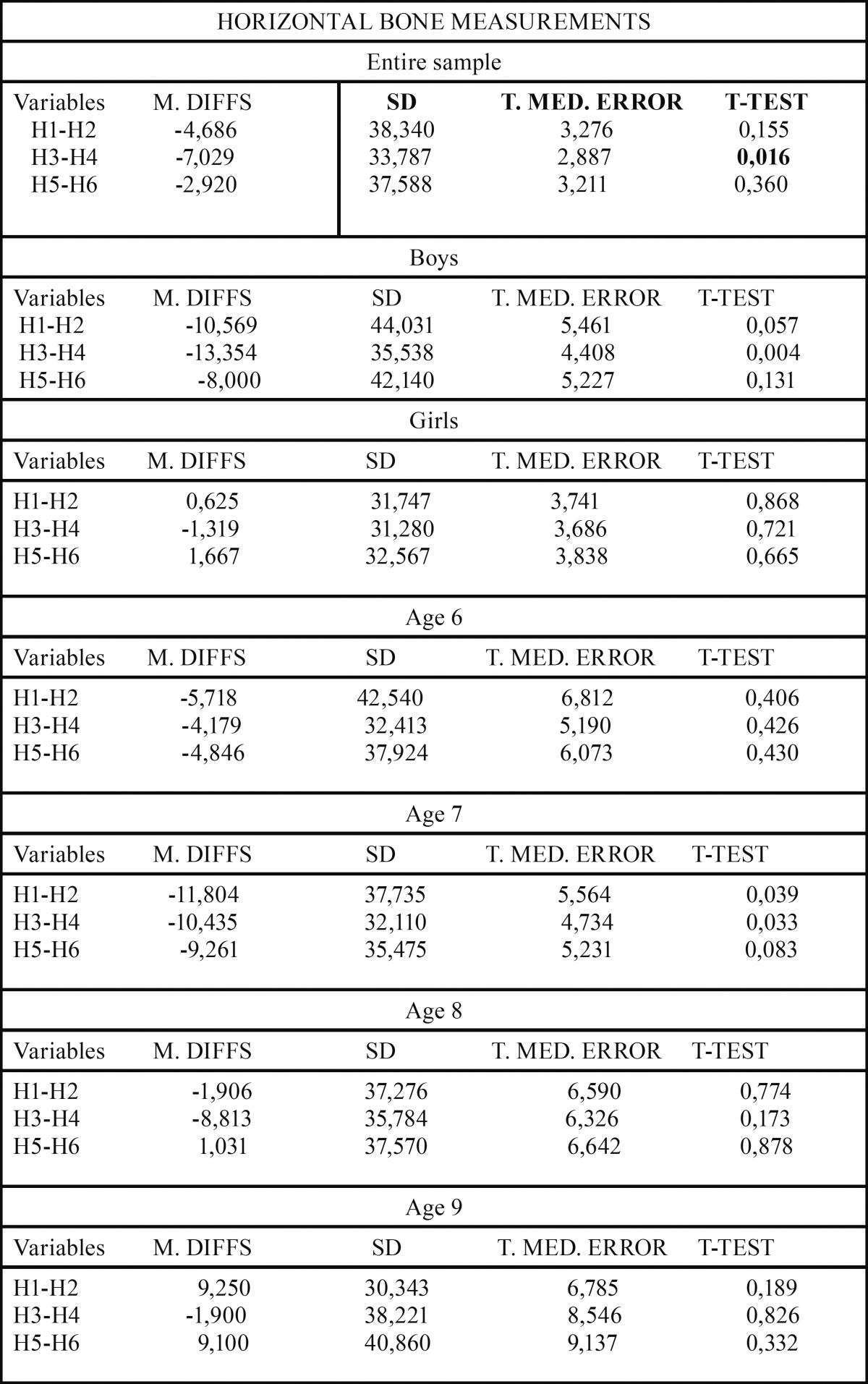
Comparative analysis of the symmetry of horizontal measurements in the entire sample with UPC. M. DIFFS: Median diffs; SD: Standard Deviation; T. MED. ERROR: Standard median error; T-TEST: Significance.

## Discussion

There are few studies in the literature that evaluate asymmetry and possible quantifiable skeletal changes at an early age using routine x-ray projections such as orthopantomography (10,21). Some research done on children does not specify their real age (10,15).

As for the use of horizontal, vertical, oblique or angular metric variables, and the method employed, there is no agreement among the different authors (14,16,19,21-22). The most widely-used method is the one proposed by Habets *et al.* (20).

Kiki *et al.*. determined the degree of condylar asymmetry in a sample of patients aged from 11 to 17, using vertical measurements traced on the orthopantomograph. Unlike our study, the patients with bilateral posterior crossbite did not present statistically significant differences between the left and right sides (14).

Kilic *et al.* determined the degree of mandibular asymmetry in a paediatric population with unilateral posterior crossbite. They also used the method described by Habets *et al.* After analysing the results, they observed a certain degree of condylar asymmetry in the patients with malocclusion (15).

Uysal *et al.* studied mandibular asymmetry in patients with unilateral and bilateral posterior crossbite. The median age was 13.06+/-3.52 years. Unlike our study, they used vertical measurements. In their results, they did not in any case find statistically significant differences between the two sides (21).

Durval *et al.* analysed, as we did in our study, mandibular asymmetry in children with unilateral posterior crossbite. They used horizontal, vertical and angular variables. They found diverging results on the crossbite and the non-crossbite sides in the measurements of mandibular length and the position of the condyles10. In our study, using only horizontal variables, we only found significant differences between the left and right variables (H1-H2 and H3-H4), with respect to the size of the mandibular body.

Given the scarcity of studies analysing mandibular symmetry through the use of horizontal metric variables traced on panoramic x-rays, we find it difficult to compare our results with those of other, similar research projects.

In the total sample, in the group of boys aged 6 and 7, we observed larger mandibular dimensions on the side without malocclusion. However, in the sample of girls aged 8 and 9, some of the variables corresponding to the side with malocclusion were greater, although on this occasion no significance was found. We can say that age and sex can alter bone size, keeping in mind the study variables.

## Conclusions

In our study, differences were observed in the size of the horizontal measurements, keeping in mind the side with the malocclusion. Some of these differences were significant as a function of the sex and age of the study sample.

Although efficient and precise diagnostic methods exist, panoramic x-rays, because they are a habitual and very accessible record, continue to be used frequently in the diagnosis of different pathologies, even though analysis of the tracing of linear and angular measurements does not represent a real projection of the object under study. However, it might be of help in diagnosing possible mandibular bone changes in patients with unilateral posterior crossbite.
